# Clinical efficacy of synbiotics in children with allergic rhinitis: An observational cohort study from a private medical center in Peru

**DOI:** 10.1002/iid3.736

**Published:** 2022-11-16

**Authors:** Cesar A. Galvan Calle, Cecilia Díaz Vásquez, Ricardo Muñoz Leon, Edgar E. Matos Benavides, Alejandra V. Verde Leon

**Affiliations:** ^1^ EMEDIC Salud Lima Peru; ^2^ Instituto Nacional de Salud del Niño Lima Peru; ^3^ Instituto Nacional de Salud del Niño San Borja Lima Peru; ^4^ Hospital San Bartolome Lima Peru

**Keywords:** allergic rhinitis, child, Peru, synbiotics, treatment outcome

## Abstract

**Background:**

Probiotics in allergic rhinitis (AR) have shown improvement in clinical and quality of life scores, whereas the role of synbiotics in the treatment of AR has been poorly investigated. The purpose of this study was to evaluate the clinical efficacy of synbiotics in children with AR.

**Methods:**

An observational, prospective cohort study of pediatric outpatients with AR from a private medical center in Peru (2021) was conducted. At baseline, patients who were prescribed synbiotics during routine and those who were not (controls) recruited and followed up on Days 30, 60, and 90 of follow‐up. Clinical efficacy was assessed with differences in Visual Analogous Scale (VAS), Total Nasal Symptom Score (TNSS), Rhinitis Control Assessment Test (RCAT), and the Pediatric Rhinoconjunctivitis Quality of Life Questionnaire (PRQLQ) scores between groups at follow‐up. Mean differences ± standard deviation (SD) and 95% confidence intervals (95% CI) are reported.

**Results:**

Two hundred and fifteen participants were analyzed. Compared to controls (*n* = 115), those who used synbiotics (*n* = 100) had significantly lower VAS (mean difference 1.3; 95% CI: 0.8–1.8), TNSS (mean difference 1.1; 95% CI: 0.5–1.7) and higher RCAT scores and PRQLQ scores (mean difference 2.2; 95% CI: −3.3 to −1.2) and (mean difference 7.0; 95% CI: 3.1–10.9), respectively, at Day 90 of follow‐up

**Conclusions:**

This paper reports significant improvement in clinical (VAS, RCAT, TNSS) and quality of life (PRQLQ) scores of small and large sizes, respectively. These preliminary findings support the need of future trials to assess the role of synbiotics in children with AR.

## Graphical abstract

Our results supported a preliminary improvement in clinical scores (VAS, RCAT, TNSS), and a significant improvement in quality of life (PRQLQ) scores above minimal important differences. These findings show the need of conducting future trials to evaluate the efficacy of synbiotics in children with AR.

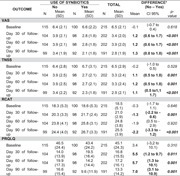

## 1 INTRODUCTION

Allergic rhinitis (AR) affects up to 20%–25% of children worldwide,^1^, ^2^ in addition to showing a trend of increasing prevalence over the last years.^2^

A group of patients with AR (e.g., of moderate or severe disease severity) do not achieve control with standard therapy alone and may benefit from add‐on treatment with probiotics, prebiotics, or synbiotics. In synbiotics both pre‐ and probiotics act synergistically to regulate the intestinal immune response related to allergic diseases; thus, providing a basis to be used in the prevention and treatment of AR.^3^

Several systematic reviews have shown that probiotics have a safety profile,^4^, ^5^ and although they may not prevent incident AR in childhood (if used earlier in life),^6^, ^7^, ^8^ when used as a treatment in patients with AR, probiotics might improve their symptoms burden, disease control, disease severity, and quality of life.^9^, ^10^ Nonetheless, synbiotics have been less investigated^11^: a few number of studies have found that synbiotics administration in AR improved symptoms,^5^ quality of life,^12^ and interleukin‐17 levels.^13^ Thus, more contributing research is needed to assess the benefits of synbiotics in AR.

Therefore, the purpose of this prospective observational study was to evaluate the clinical efficacy of synbiotics in children with AR in a private medical center in Lima, Peru.

## 2 METHODS

### 2.1 Study design

We conducted a prospective cohort study of pediatric outpatients with AR from a private medical center located in Lima, Peru, between February 2021 and November 2021. At baseline, a group of patients receiving synbiotics (as part of their routinary care) and a group who did not (controls) were recruited; then, they were followed up monthly up to 3 months later.

### 2.2 Sampling and sample size

Patients were recruited consecutively as they were identified after their routinary medical outpatient consultations. The sample size was calculated with Epidat® v.4.2, considering a potency of 80% with a minimum mean difference to detect of 1.1 for the Total Nasal Symptom Score (TNSS) between groups, a standard deviation of 2.7, and a confidence level (CI) of 95%.

### 2.3 Statistical analysis

The data analysis considered the assessment of the difference in means of the result variables through the application of the *T*‐test for independent samples. The analysis was performed in the statistical software IBM SPSS version 26 with a significance level of .05.

### 2.4 Ethics

The protocol of this study was approved by the Ethics Committee of the Hospital Nacional Madre Niño San Bartolomé (reference number 01094‐21, Supporting Information: Appendix 1).

### 2.5 Procedures

A summary of study procedures is shown in Table 1. All enrolled patients were verified to receive the appropriate doses according to local guidelines. Patients receiving synbiotics were instructed not to consume drinks or foods with probiotics or prebiotics, a list of such products was provided.

**Table 1:** Study procedures

Procedures	Interview			
	First (baseline)	Second (Day 30)	Third (Day 60)	Fourth (Day 90)
Setting of the interview				
At office	X			
Remote (Zoom meeting)		X	X	X
Clinical				
Medical chart review	X	X	X	X
Clinical interview	X	X	X	X
Total Nasal Symptom Score (TNSS)	X	X	X	X
Disease severity (ARIA)	X	X	X	X
Visual analogous scale (VAS)	X	X	X	X
Control asthma allergic rhinitis test (CARAT)	X	X	X	X
Rhinitis Control Assessment Test (RCAT)	X	X	X	X
Pediatric Rhinoconjunctivitis Quality of Life Questionnaire (PRQLQ)	X	X	X	X

### 2.6 Exposure variables

The product of exposure was the synbiotics, we use BagoVital Inmune® that is compounded by *Lactobacillus acidophilus Rosell‐52*, *Bifidobacterium infantis Rosell‐33*, *Bifidobacterium bifidum Rosell‐71* (5 × 10^9^ colony forming units) fructooligosaccharides 750 mg and Vitamin C 12 mg and is free of sale. Synbiotics were indicated before recruitment by the attending physician during regular medical appointments.

### 2.7 Outcome variables

The severity of AR symptoms, measured by changes in Visual Analogous Scale (VAS) total scores between exposure groups at follow‐up; the intensity of AR symptoms, measured by changes in total symptoms score total scores between exposure groups at follow‐up; the control of AR symptoms, measured by changes in Rhinitis Control Assessment Test (RCAT) total scores between exposure groups at follow‐up; and quality of life, measured by changes in the Pediatric Rhinoconjunctivitis Quality of Life Questionnaire (PRQLQ) total scores between exposure groups at follow‐up.

## 3 RESULTS

### 3.1 Baseline description of the total sample

Data from a total of 215 participants, with a mean (SD) age of 10.4 (3.7), and 55.4% of males were analyzed (baseline characteristics are shown in Table 2). The most frequent comorbidity was allergic conjunctivitis (74.4%). Regarding family history, most patients had a first‐degree relative with allergic rhinoconjunctivitis (78.2%).

**Table 2:** Baseline characteristics of patients with allergic rhinitis from a private medical center in Lima, Peru (2021)

Characteristics	Use of synbiotics		Total
	No	Yes	
	*N* = 115 (%)	*N* = 100 (%)	*N* = 215 (%)
Age (years) (mean ± standard deviation)	10.4 ± 3.6	10.4 ± 3.8	10.4 ± 3.7
Sex			
Female	57 (49.6)	39 (39.0)	96 (45.6)
Male	58 (50.4)	61 (61.0)	119 (55.4)
Asthma			
No	42 (36.5)	30 (30.0)	72 (33.5)
Yes	73 (63.5)	70 (70.0)	143 (66.5)
Allergic conjunctivitis			
No	27 (23.5)	28 (28.0)	55 (25.6)
Yes	88 (76.5)	72 (72.0)	160 (74.4)
Atopic dermatitis			
No	61 (53.1)	56 (56.0)	117 (54.4)
Yes	54 (46.9)	44 (44.0)	98 (45.6)
Drug allergy			
No	75 (65.2)	71 (71.0)	146 (67.9)
Yes	40 (34.8)	29 (29.0)	69 (32.1)
Food allergy			
No	97 (84.3)	93 (93.0)	190 (88.4)
Yes	18 (15.7)	7 (7.0)	25 (11.6)
Family history of asthma			
No	49 (42.6)	23 (23.0)	72 (33.5)
Yes	66 (57.4)	77 (77.0)	143 (66.5)
Family history of allergic rhinoconjunctivitis			
No	22 (19.1)	25 (25.0)	47 (21.8)
Yes	93 (80.9)	75 (75.0)	168 (78.2)
Family history of atopic dermatitis			
No	51 (44.3)	54 (54.0)	105 (48.8)
Yes	64 (55.7)	46 (46.0)	110 (51.2)
Family history of drug allergy			
No	100 (87.0)	79 (79.0)	179 (83.3)
Yes	15 (13.0)	21 (21.0)	36 (16.7)
Family history of food allergy			
No	103 (89.6)	96 (96.0)	199 (92.6)
Yes	12 (10.4)	4 (4.0)	16 (7.4)

Clinical characteristics related to AR are shown in Table 3.

**Table 3:** Clinical characteristics of patients with allergic rhinitis (AR) from a private medical center in Lima, Peru (2021)

Characteristics	Use of synbiotics		Total
	No	Yes	
	*N* = 115 (%)	*N* = 100 (%)	*N* = 215 (%)
Type of AR			
Persistent	85 (73.9)	87 (87.0)	172 (80.0)
Intermittent	30 (26.1)	13 (13.0)	43 (20.0)
Severity of AR (ARIA)			
Mild	55 (47.8)	45 (45.0)	100 (46.5)
Moderate	60 (52.2)	55 (55.0)	115 (53.5)
Use of antihistaminic			
No	33 (28.7)	25 (25.0)	58 (27.0)
Yes	82 (71.3)	75 (75.0)	157 (73.0)
Use of inhaled corticosteroids			
No	94 (81.7)	70 (70.0)	146 (67.9)
Yes	21 (18.3)	30 (30.0)	51 (23.7)

### 3.2 Comparison by use of synbiotics

Of the 215 participants, 100 (46.5%) used synbiotics and 115 (53.5%) not. Compared to patients who did not use synbiotics, participants who used them had a higher proportion of males (61.0% vs. 50.4%), family history of asthma (77.0% vs. 57.4%), persistent AR (87% vs. 73.9%), and of use of inhaled corticosteroids (30.% vs. 18.3%). The differences in the proportion of resting characteristics between exposure groups were below 10%.

### 3.3 Evaluation of clinical efficacy

Results of the association between synbiotics use and the outcomes of clinical efficacy are presented in Table 4. At baseline, there were no differences in the VAS, TNSS, RCAT, and PRQLQ scores between those who used synbiotics and those who not. At Month 3 of follow‐up, synbiotics use was associated with improved disease severity, intensity of symptoms, and quality of life: the VAS, TNSS, and PRQLQ scores were significantly lower by 1.3 points (95% CI:  0.8–1.8), 1.1 points (95% CI: 0.5–1.7), and 7.0 points (95% CI: 3.1–10.9), accordingly, among those who received synbiotics compared to controls. The RCAT score after 3 months of follow‐up was significantly higher by 2.2 points (95% CI: −3.3 to −1.2) in those who received synbiotics (vs. controls).

**Table 4:** Clinical efficacy of synbiotics use in patients with allergic rhinitis from a private medical center in Lima, Peru (2021)

Outcome	Use of synbiotics				Total		Difference^a^ (No − Yes)		
	No		Yes						
	*N*	Mean (SD)	*N*	Mean (SD)	*N*	Mean (SD)	Mean	95% CI	*p* Value
VAS									
Baseline	115	6.4 (2.1)	100	6.6 (2.2)	215	6.5 (2.1)	−0.1	(−0.7−0.4)	.618
Day 30 of follow‐up	104	3.9 (2.1)	98	2.8 (1.8)	202	3.4 (2.0)	1.2	(0.6–1.7)	<.001
Day 60 of follow‐up	104	3.9 (2.1)	98	2.8 (1.8)	202	3.9 (2.0)	1.2	(0.6–1.7)	<.001
Day 90 of follow‐up	99	3.4 (1.9)	92	2.1 (1.8)	191	2.8 (1.9)	1.3	(0.8–1.8)	<.001
TNSS									
Baseline	115	6.4 (2.8)	100	6.7 (3.1)	215	6.5 (2.9)	−0.2	(−1.0–0.5)	.529
Day 30 of follow‐up	104	3.9 (2.5)	98	2.7 (2.1)	202	3.3 (2.4)	1.1	(0.5–1.8)	.001
Day 60 of follow‐up	104	3.9 (2.5)	98	2.7 (2.1)	202	3.3 (2.4)	1.2	(0.5–1.8)	.001
Day 90 of follow‐up	99	3.4 (2.2)	92	2.3 (1.8)	191	2.9 (2.1)	1.1	(0.5 to 1,1 1.7)	<.001
RCAT									
Baseline	115	18.3 (5.3)	100	18.6 (5.3)	215	18.5 (5.1)	−0.3	(−1.7–1.1)	.646
Day 30 of follow‐up	104	20.3 (3.3)	98	21.7 (2.4)	202	21.0 (2.9)	−1.3	(−2.2 to −0.6)	.001
Day 60 of follow‐up	104	23.8 (4.1)	98	25.8 (3.1)	202	24.8 (3.8)	−1.9	(0.5 to −2.9)	.920
Day 90 of follow‐up	99	24.4 (4.0)	92	26.7 (3.3)	191	25.5 (3.9)	−2.2	(−3.3 to −1.2)	<.001
PRQLQ									
Baseline	115	46.5 (24.4)	100	43.4 (24.2)	215	45.1 (24.3)	3.4	(–3.2–10.1)	.310
Day 30 of follow‐up	104	14.0 (13.9)	98	19.5 (16.4)	202	16.8 (15.5)	5.5	(1.2–9.7)	.011
Day 60 of follow‐up	104	19.9 (17.3)	98	14.2 (14.4)	202	17.2 (16.1)	5.7	(1.3–10.1)	.001
Day 90 of follow‐up	99	16.6 (15.4)	92	9.6 (11.9)	191	13.3 (14.2)	7.0	(3.1–10.9)	.001

Abbreviations: PRQLQ, Pediatric Rhinoconjunctivitis Quality of Life Questionnaire; RCAT, Rhinitis Control Assessment Test; TNSS, Total Nasal Symptom Score; VAS, Visual Analogous Scale.

^a^ Differences <.05 are bolded.

## 4 DISCUSSION

Our findings in this observational cohort study showed evidence supporting the preliminary clinical benefits of add‐on treatment with synbiotics in children with AR. Among those who used synbiotics compared to controls, there was a significant improvement in quality of life (PRQLQ) scores. Differences in clinical scores of disease severity (VAS), disease control (RCAT), and intensity of symptoms (TNSS) were also significant but in less proportion. These findings may guide further assessments of the efficacy of synbiotics in children with AR.

These study results contribute to the scarce previous evidence evaluating synbiotics treatment in patients with AR.^3^ Jalali et al.^12^ conducted a crossover RCT in Iran (2015) among patients with persistent AR (*n* = 152, mean age ± SD: 30.1 ± 7.6); they compared budesonide + probiotic (seven different gram‐positive organisms + fructooligosaccharide) versus budesonide + placebo, finding significantly improvements in quality of life (physical and mental components of the Short Form 36‐Item Health Survey: Cohen effect sizes = 0.40 and 0.33, respectively), and clinical scores (sinonasal outcome test 22: Cohen effect size = 1.31; Control of Allergic Rhinitis and Asthma Test: Cohen effect size = 1.14). Dehnavi et al.^13^ conducted a randomized controlled trial (RCT) among patients with AR (mean age ± SD: 24.0 ± 12.8) in Iran (2016); they compared immunotherapy + synbiotic by 2 months (*Streptococcus thermophilus*, *Bifidobacterium* spp., *Lactobacillus* spp., and fructooligosaccharide; *n* = 8) versus placebo + immunotherapy (*n* = 9), showing no significant differences in clinical symptoms (sinonasal outcome test 22) or quality of life (mini‐Rhinoconjunctivitis Quality of Life Questionnaire), but a significant decrease in interleukin‐17 levels at 6 months of follow‐up. Last, daily administration of synbiotics for 6 months (*Lactobacillus rhamnosus*, *Bifidobacterium breve*, *Propionibacterium freudenreichii*, galacto‐oligosaccharides) to newborn infants from pregnant mothers (who received the same probiotics before childbirth) did not reduce the incidence of allergic diseases (including AR) in an RCT from Finland (2000–2003) by Kukkonen et al.^14^

Previous RCTs^12^, ^13^, ^14^ show the need to standardize target populations (of higher sample sizes), the type of synbiotic used, the outcomes of interest, and the length of follow‐up in the assessment of synbiotics for the prevention or treatment of AR. Besides, our findings of improved clinical (TNSS, VAS, and RCAT) and quality of life scores (PRQLQ) with synbiotics treatment may be compared with minimal important differences for each instrument and with studies using probiotics only.

In our study, synbiotics caused a significant improvement of clinical scores: VAS (mean difference 1.3; 95% CI: 0.8–1.8), TNSS (mean difference 1.1; 95% CI: 0.5–1.7) and RCAT scales (mean difference 2.2; 95% CI: −3.3 to −1.2) were significantly lower among those who received synbiotics (vs. controls). In the case of probiotics, a metanalysis (2016)^10^ showed clinical benefits of their use in 77% (*n* = 17/22) of RCTs. Also, probiotics reduced significantly nasal and ocular symptoms score (mean standard difference: −1.23 and −1.8, respectively). More recent metanalysis (2022)^15^, ^16^, ^17^ confirmed at least a small and heterogeneous clinical benefit with synbiotics.

A significant improvement in quality of life was associated with synbiotics in our study: PRQLQ score was significantly higher (mean difference 7.0; 95% CI: 3.1–10.9) among children who used synbiotics (vs. controls); this difference was large and above the minimal important difference of 0.5. In the case of probiotics, the same meta‐analysis (2016)^10^ associated their use with significant improvement in the total quality of life scores (mean standard difference: −1.84).^10^

However, our study has limitations related to the observational design and the prescription bias may be present since there were relevant differences between synbiotic and control groups. Additionally, even though this study has a control group a placebo effect may be present. Our findings may inform future studies to define the role of synbiotics in the context of AR.

## AUTHOR CONTRIBUTIONS

Cesar A. Galvan Calle, Cecilia Díaz Vásquez, Ricardo Muñoz Leon, Edgar E. Matos Benavides, and Alejandra V. Verde Leon were involved in the same proportion in the design of the study, collection of clinical data, redaction, and revision of the paper.

## CONFLICT OF INTEREST

Cesar Alberto Galvan Calle has received a research grant from Laboratorio Bago. Honorarios as a speaker from Laboratorio Bago and Laboratorio Mead Johnson Nutritional. The remaining authors declare no conflict of interest.

## Supporting information

Supporting information.

## Data Availability

The data that support the findings of this study are available from the corresponding author upon reasonable request.

## References

[1] Aït‐Khaled N , Pearce N , Anderson HR , et al. Global map of the prevalence of symptoms of rhinoconjunctivitis in children: the International Study of Asthma and Allergies in Childhood (ISAAC) phase three. Allergy. 2009;64(1):123‐148. 19132975 10.1111/j.1398-9995.2008.01884.x

[2] Nur Husna SM , Tan HTT , Md Shukri N , Mohd Ashari NS , Wong KK . Allergic rhinitis: a clinical and pathophysiological overview. Front Med. 2022;9:874114. 10.3389/fmed.2022.874114 PMC9021509 35463011

[3] Meirlaen L , Levy EI , Vandenplas Y . Prevention and management with pro‐, pre and synbiotics in children with asthma and allergic rhinitis: a narrative review. Nutrients. 2021;13(3):934. 33799367 10.3390/nu13030934 PMC7999316

[4] West CE , Dzidic M , Prescott SL , Jenmalm MC . Bugging allergy; role of pre‐, pro‐ and synbiotics in allergy prevention. Allergol Int. 2017;66(4):529‐538. 28865967 10.1016/j.alit.2017.08.001

[5] Bergmann KC , Krause L , Hiller J , et al. First evaluation of a symbiotic food supplement in an allergen exposure chamber in birch pollen allergic patients. World Allergy Organ J. 2021;14(1):100494. 33376575 10.1016/j.waojou.2020.100494 PMC7753943

[6] Peng Y , Li A , Yu L , Qin G . The role of probiotics in prevention and treatment for patients with allergic rhinitis: a systematic review. Am J Rhinol Allergy. 2015;29(4):292‐298. 26163249 10.2500/ajra.2015.29.4192

[7] Du X , Wang L , Wu S , et al. Efficacy of probiotic supplementary therapy for asthma, allergic rhinitis, and wheeze: a meta‐analysis of randomized controlled trials. Allergy Asthma Proc. 2019;40(4):250‐260. 31262380 10.2500/aap.2019.40.4227

[8] Savilahti E . Probiotics in the treatment and prevention of allergies in children. Biosci Microflora. 2011;30(4):119‐128. 25045317 10.12938/bifidus.30.119 PMC4103638

[9] Chen N , Liu F , Gao Q , Wang R , Zhang L , Li Y . A meta‐analysis of probiotics for the treatment of allergic airway diseases in children and adolescents. Am J Rhinol Allergy. 2022;36(4):480‐490. 10.1177/19458924221080159 35238209

[10] Güvenç IA , Muluk NB , Mutlu FŞ , et al. Do probiotics have a role in the treatment of allergic rhinitis? A comprehensive systematic review and meta‐analysis. Am J Rhinol Allergy. 2016;30(5):e157‐e175. 10.2500/ajra.2016.30.4354 27442711

[11] Dykewicz MS , Wallace DV , Amrol DJ , et al. Rhinitis 2020: a practice parameter update. J Allergy Clin Immunol. 2020;146:721‐767. 32707227 10.1016/j.jaci.2020.07.007

[12] Jalali MM , Soleimani R , Alavi Foumani A , Ganjeh Khosravi H . Add‐on probiotics in patients with persistent allergic rhinitis: a randomized crossover clinical trial. Laryngoscope. 2019;129(8):1744‐1750. 30794334 10.1002/lary.27858

[13] Dehnavi S , Azad F , Hoseini R , et al. A significant decrease in the gene expression of interleukin‐17 following the administration of synbiotic in patients with allergic rhinitis who underwent immunotherapy: a placebo‐controlled clinical trial. J Res Med Sci. 2019;24:51. 31333730 10.4103/jrms.JRMS_543_18 PMC6611180

[14] Kukkonen K , Savilahti E , Haahtela T , et al. Probiotics and prebiotic galacto‐oligosaccharides in the prevention of allergic diseases: a randomized, double‐blind, placebo‐controlled trial. J Allergy Clin Immunol. 2007;119(1):192‐198. 17208601 10.1016/j.jaci.2006.09.009

[15] Farahmandi K , Mohr AE , McFarland LV . Effects of probiotics on allergic rhinitis: a systematic review and meta‐analysis of randomized clinical trials. Am J Rhinol Allergy. 2022;36(4):440‐450. 10.1177/19458924211073550 35099301

[16] Yan S , Ai S , Huang L , et al. Systematic review and meta‐analysis of probiotics in the treatment of allergic rhinitis. Allergol Immunopathol. 2022;50(3):24‐37. 10.15586/aei.v50i3.507 35527653

[17] Luo C , Peng S , Li M , Ao X , Liu Z . The efficacy and safety of probiotics for allergic rhinitis: a systematic review and meta‐analysis. Front Immunol. 2022;13:848278. https://www.frontiersin.org/article/10.3389/fimmu.2022.848279 10.3389/fimmu.2022.848279 PMC9161695 35663980

