# Supplementary material for: Clinical efficacy of synbiotics in children with allergic rhinitis: An observational cohort study from a private medical center in Peru
Source: Immun Inflamm Dis. 2022 Nov 16;10(12):e736. doi: 10.1002/iid3.736 (PMC9667200; doi:10.1002/iid3.736)
Supplement: Supplementary file 1 — Supporting information. [file IID3-10-e736-s001.pdf]

"Año del Bicentenario del Perú: 200 años de Independencia"

Lima, 12 abril de 2021

OFICIO N°151-2021-OADI-HONADOMANI-SB

ALEJANDRA VANESA VERDE LEÓN
Investigadora Principal
Presente.-

Expediente N°01094-21

Tengo el agrado de dirigirme a usted para saludarla cordialmente y en relación al Proyecto de Investigación titulado:

"EFECTO CLÍNICO DEL USO DE SIMBIÓTICOS EN NIÑOS CON RINITIS ALÉRGICA EN UN CENTRO DPRIVADO DE LIMA, 2021"

Al respecto se informa lo siguiente:

El planteamiento del tema, la metodología estadística propuesta, así como el plan de análisis de los resultados a obtener son apropiados para el estudio

Conclusión:

El Comité Investigación del HONADOMANI San Bartolomé y el Comité Institucional de Ética en Investigación, aprueban de manera expedita el proyecto de Investigación con Expediente N°01094-21

Hago propicia la oportunidad para renovar los sentimientos de nuestra consideración y estima personal.
atentamente

HONADOMANI – DIRECCIÓN DE APOYO A LA DOCENCIA E INVESTIGACIÓN – SAN BARTOLOMÉ

MINISTERIO DE SALUD
Hospital Nacional Docente Madre Niño
"SAN BARTOLOMÉ"
MC. HUGO DELGADO BARTRA
Jefe de la Oficina de Apoyo a la Docencia e Investigación

HOSPITAL SAN BARTOLOMÉ - HONADOMANI – COMITÉ DE ÉTICA EN INVESTIGACIÓN – LIMA - PERÚ

HDB/vma
cc.archivo
